# The effectiveness of a health promotion with group intervention by clinical trial. Study protocol

**DOI:** 10.1186/1471-2458-12-209

**Published:** 2012-03-19

**Authors:** Maria-Antonia Campo Osaba, José-Luis Del Val, Carolina Lapena, Vicencia Laguna, Araceli García, Olga Lozano, Ziortza Martín, Rómulo Rodriguez, Enriqueta Borrás, Francesc Orfila, María Teresa Tierno

**Affiliations:** 1 Basic Area of Health Sanllehy, Catalan Institute of Health, Av. Mare de Déu de Montserrat, 16-18, 08024 Barcelona, Spain; 2 USR Barcelona. IDIAP Jordi Gol, St Elies 42, 08006 Barcelona, Spain

## Abstract

**Background:**

The promotion of health and the interventions in community health continue to be one of the pending subjects of our health system. The most prevalent health problems (cardiovascular diseases, cancer, diabetes...) are for the most part related to life habits. We propose a holistic and integral approach as the best option for tackling behavior and its determinants. The research team has elaborated the necessary educational material to realize group teaching, which we call "Health Workshops". The goal of the present study is to evaluate the effectiveness of these Health Workshops in the following terms: Health Related Quality of Life (HRQOL), incorporate and maintain a balanced diet, do physical activity regularly, maintain risk factors such as tension, weight, cholesterol within normal limits and diminish cardiovascular risk.

**Methods/Design:**

Controlled and random clinical testing, comparing a group of persons who have participated in the Health Workshops with a control group of similar characteristics who have not participated in the Health Workshops.

Field of study: the research is being done in Health Centers of the city of Barcelona, Spain.

Population studied: The group is composed of 108 persons that are actually doing the Health Workshops, and 108 that are not and form the control group. They are assigned at random to one group or the other.

Data Analysis: With Student's t-distribution test to compare the differences between numerical variables or their non parametric equivalent if the variable does not comply with the criteria of normality. (Kolmogorov-Smirnof test). Chi-square test to compare the differences between categorical variables and the Logistic Regression Model to analyze different meaningful variables by dichotomous analysis related to the intervention.

**Discussion:**

The Health Workshop proposed in the present study constitutes an innovative approach in health promotion, placing the emphasis on the person's self responsibility for his/her own health.

The rhythm of a weekly session during 8 weeks with recommended activities to put into practice, as well as the support of the group is an opportunity to incorporate healthy habits and make a commitment to self-care. The sheets handed out are a Health Manual that can always be consulted after the workshop ends.

**Trial registration:**

Clinical Trials.gov Identifier: NCT01440738

## Background

The promotion of health and the interventions in community health continue to be one of the pending subjects of our health system [1,2]. The most common health problems (heart disease, cancer, diabetes...) are related in a large amount to behavior [3-5]. The estimates are that 35% of cancers are diet related [6-8]. The increase of physical activity produces a reduction in the risk of chronic illnesses, like cardiovascular ones, arterial hypertension, type 2 diabetes mellitus and obesity, all of them associated to a sedentary life style [9]. Also, regular physical exercise helps improve mild depressions and anxieties. Some studies have objected that interventions to improve behavior or habits by changing them to healthier ones have resulted in short lived improvements[10-12]. In a systematic revision [13] in diabetes mellitus patients, they have found evidence that group education has had a positive effect for a better metabolic control, the decrease of arterial pressure and overweight, the increase of self control, the quality of life, the development of self care abilities as well as satisfaction with the treatment received, and permits verification of the effectiveness of groups in the incorporation of healthy habits, but that these positive changes do not continue after a time, once the group activity has finished. To continue with this revision and on reflection of the possible reasons of why these positive changes do not continue after a time we find that these studies about health promotion usually deal with specific aspects (physical activity, diet...) [14,15] but do not include other aspects that have influence in behavior and health and well-being.

The biomedical system has a relevant role when approaching ailments in our western society. In spite of the fact that the research team also forms part of the same biomedical system which tends to fraction and specialize, our proposal is centered on a holistic and integrating approach as the best way to tackle health behaviors and its determinants. The proposal is based on Virginia Henderson's model of the concept of nursing [16]; this model has resulted useful in individual caring, and our hypothesis is that this holistic focusing is equally useful to guide other actions of health promotion, as it contemplates the person as a whole giving importance to physical aspects as well as psychical, emotional, cultural and spiritual ones.

Henderson defines the person as whole that has 14 basic activities or requisites to be satisfied in order to maintain a person's health and well being, and develop all his/her capacities. These 14 components are:

Breathe normally, Eat and Drink Adequately, Eliminate Body Waste, Maintain Desirable Positions, Sleep and Rest, Wear Suitable Clothes, Maintain Body Temperature by adjusting clothes and modifying environment, Keep the Body clean and well-groomed, Avoid Dangers and Injuring Others, Communicate with others, express emotions, needs and fears, Worship according to one's faith, Work to have a sense of Accomplishment, Play and Participate in various forms of Recreation, Learn, discover and Satisfy Curiosity. According to this model, everybody is capable of carrying out these basic activities by themselves, and if they don't, it is because of lack of the knowledge, will, or strength to do so.

In the year 2008 the research team developed the necessary educational material to carry out group education which we named "HEALTH WORKSHOPS" [17] addressed to the general public. The workshops consist of 8 sessions with weekly cadence and gather those 14 components described by Henderson. In the different sessions we incorporate tools in order to help integrate healthy habits to daily life and improve the quality of living perceived and have a direct relation to their prevalent health problems. Also these healthy habits are contained in health system plans of all the Autonomous Communities in Spain, and are recommended in the Program of Health Prevention and Health Promotion Activities. (PAPPS) [18]

In spite of not being standard, the measurements of Heath Related Quality of Life (HRQOL), have proved valid and reliable in evaluating health results [19]. Clinical personnel and managers have recognized the importance of measuring the HRQOL as useful in informing about the most effective patient handling and in decision making in matters of health [20]. These measurements include health profiles in which the general questionnaire of Medical Outcomes Study 36 item Short Form Health Survey (SF-36) stands out, containing 36 items that cover two areas, the functional state (4 physical dimensions) and the emotional well-being (4 mental dimensions) [19]. Furthermore this questionnaire has normative population values for the Spanish population [21,22] for which reason we consider using it as the main variable in the study.

## Hyphotesis

The Group Intervention of Health Promotion that this study proposes (which contemplates in an integral way biological, physiological, social, cultural and spiritual aspects of the person related to health and well-being), improves the quality of life related to health (HRQOL) and facilitates the incorporation and keeping of healthy habits.

### Objectives

#### Principal objective

Assess the effectiveness of the Health Workshops, in terms of HRQOL measured by the SF-36 Questionnaire.

Secondary objectives:

1.) To know the effectiveness of the Health Workshops in relation to:

• Incorporating and maintaining a balanced diet.

• Keeping up a regular physical activity.

• Maintaining risk factors (Blood Pressure, Weight, and Cholesterol) within normal limits.

• Risk reduction cardiovascular disease (CVD)

2.) To make an estimate of the costs of the Health Workshop.

## Method/Design

Design: Cluster randomized trial, controlled and multicentric, comparing one group of patients that take part in the Health Workshops, with another group of similar characteristics that don't.

Setting: The study will be done in Heath Care Centers of Barcelona City.

Randomization unit: Primary care Teams (PCT)

Study Population: Population around the Centers that participate in the Study and that meet the criteria of inclusion.

Inclusion criteria:

• Persons of both sexes between 18 and 65 who consent to do so.

Exclusion criteria:

• If there is an objection to participating during a period of 12 months.

• If there a language difficulty, unable to understand or express in Spanish or Catalan.

• If there are acute mental problems.

• Other health problems which may be a difficulty in following the workshop, (like terminals, organic pathology...).

Sample size calculation: Accepting an Alfa risk of 0.05 and a Beta risk of 0.2 in a bilateral contrast, 108 individuals per group are needed in order to detect how statistically effective it is in a 20% difference between groups in quality of life with the SF-36 Questionnaire. We have estimated a 10% rate of loss in participation. The ARCOSENO approximation has been used.

Variables in the study: We collect socio-demographic variables from all participants: age, sex, studies, employment or work situation.

Principal Variable: HRQOL measured by means of SF-36 Questionnaire. We have selected the quality of life perceived indicator as the main variable as it is sensible to small changes [19].

Variables related to the intervention:

• Number of sessions the person has attended.

• IPAC Questionnaire for variables related to physical activity (the IPAC questionnaire has been used in research around this area so it allows us to compare the data obtained.)

• The variables related to eating habits will be collected by means of Questionnaire PREDIMED [15], which is validated and used in our area, as so allow us to compare results.

• Blood Pressure, Lipid Profile, Height, Weight, will be gathered from the computerized clinical history.

• To estimate the costs we will add the time spent by professionals directly related with the intervention (telephone calls, meetings with participants, gathering of data, workshops...)

• Gathering of information: the gathering of data will be done in both groups, (intervention and control groups): before each intervention (Health Workshop), at the end of the intervention and in 6 months and 12 through self administered questionnaires in Teleform^© ^format.

**Figure 1:**
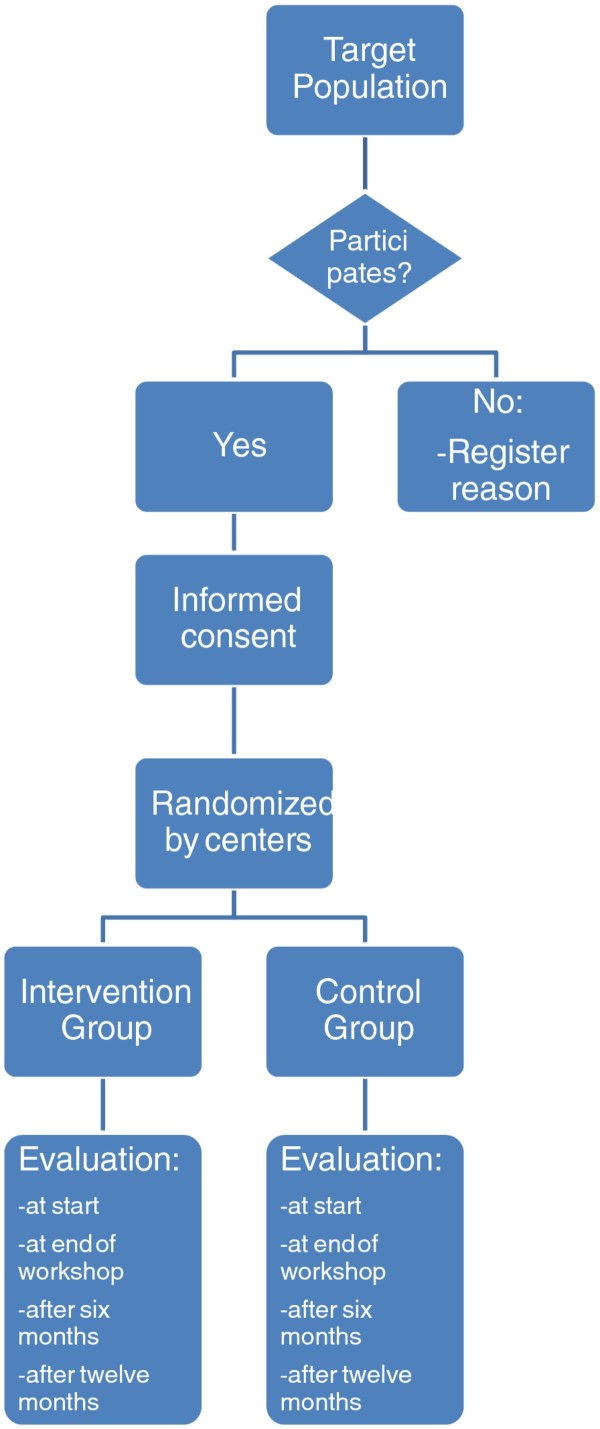
**Algorithm of the study**.

Description of the Study (Figure 1)

The stages of this study are the following:

Phase Recruitment and selection:

1 The recruitment of the PCT: The project is explained to professionals belonging to the Health Centre. Those interested in participating in the recruiting of patients will sign a commitment of participation in the Project. The participants will be recruited by these professionals and their inclusion will be consecutive. Once the recruitment has ended, the groups will be randomized in the intervention or control group. Persons interested will fill up an application form for the Workshop, which will be available in their Health Care Center (HCC) and where they have to give their name, phone number, and time availability.

The Blood Pressure (BP) and Weight data will be gathered (in those persons who consent), at an individual visit after the informative meeting. They will be asked to have a Lipid Profile (if it is not available in their clinical history in a 3 months period before the workshops begins), and again after 12 months.

Phase The Forming of the Study Groups

2 Based on the persons who have given their consent to participate, an random assignation to one or another group will take place, and the workshops will then start (with groups of 12 to 15 persons in each).

Phase Intervention (only in the group intervened)

3 The group intervention then starts in 8 sessions in which life activities will be approached in a progressive manner. During the sessions, tools will be offered to increase knowledge and develop abilities and motivations that help with life activities and the progressive incorporation of healthy habits. At the end of each session support material will be handed out with the session's contents and indications of activities to practice during the week.

The workshops will be offered at different schedules to facilitate assistance. The contents of the sessions are in Table 1.

**Table 1:** Subject matter of the sessions in the work shop

Session	Content
1	Breathing. Tobacco, learning from experience.

2.	Eating and drinking adequately, a balanced diet.

3.	Elimination. The importance of regular disposal. Guides to avoid constipation. Hygiene. The importance of personal hygiene.

4.	Movement and maintaining adequate positions. Doing physical activity. Wearing adequate clothes.

5.	Sleep and rest. Recommendations for a good rest and sleep. Leisure activities. The importance of dedication time to leisure.

6.	Avoid physical and emotional dangers. Self-esteem.

7.	Communication with others and social interaction. Managing one's emotions.

8.	Work and feel useful. The importance of activities that produce a sense of satisfaction.

Each session starts with a breathing exercise that helps participants to center themselves in the group. Next they share the week's experiences and clarify doubts. Followed by the theoretical content about the subject of the session and the practical exercises that to be done during the next week. The session ends with a space to clarify doubts and share reflections that have come up through the exposure of the theoretical concepts.

At the end of each session we hand out texts to be kept in their files and that will form, at the end of the Health Workshop, a MANUAL OF HEALTH CARE AND MAINTENANCE.

We have elaborated specific material, (with slides and points dealt with in the sessions) to ensure the unification of the message given. Also, we have elaborated support and reminder material for each session which gathers all the contents of the sessions. Such material is handed out at the end of each session to be kept in the files provided at the beginning of the Health Workshop, and which will, in the end, form a Manual for Health Self-Care and Maintenance.

The control group will continue to receive normal attention and will have the possibility, if they so desire, of doing the Health Workshops at the end of the Project, (if it has proven effective), once the 12 months are over with the last evaluation.

Phase Closing of the Project

4 Gathering and analyzing the last data and communicating the results.

### Data analysis

The analysis will be done on an intention to treat basis. Those who have done less than six sessions will be considered withdrawals.

The descriptive analysis will be done in proportion to the quality variables and the average values for the quantities, with the corresponding measures of dispersion.

To observe the effect of the intervention in quality of life related to health, the change of habits and the costs derived from related resources, the T-Tests will be used for paired data and the McNemar Test or equivalents for quantitative or qualitative variables or their non parametric equivalents, if the distribution of the variable does not comply with the criteria of normality (Kolmogorov-Smirnof).

To analyze the factors that have influence over the changes of quality of life related to health (CVRS) and those related with the intervention, models of multiple regressions will be used. In each regression model the dependent variable will be derived from the calculation or the difference between ratings in the beginning of the study and the ratings at the end of the SF-36; the rest of the variables determined in the study will be included as independent variables. As the first step in the analysis of regression the adequate diagnosis will be done to detect extreme values and verify conditions of applicability (homocedastisity, normality and independence of waste). If needed the necessary transformations will be done in the dependent variables for the fulfillment of applicability conditions. The criteria of selection of variables for the estimation of multivariable models will be a Forward-Stepwise with the criteria of input of p < 0.05 and a criteria of output of p > 0.10.

### Quality control

The proceedings used to guaranty the quality of the information are the following:

• The intervention is done by the persons in the research group who created all the educational material being used in the session, which guarantees a homogenous intervention.

• The gathering of data is done through self-operated questionnaires in Teleform^©^. This automated software that reads scanned questionnaires, performs a validating process of the data with previously established parameters, and once the data is validated these are poured into a specifically created database.

• The introduction of information or data will be performed by a person hired to provide technical assistance for this study.

### Ethical aspects

It will be an indispensable requisite to participate in this study that all participants sign their informed consent. The same document specifies that the participation has to be voluntary, with the possibility of leaving the program if he or she so desires, and that the intervention will not have a negative effect in their health care assistance. They will be given both oral and written information. The subjects will have opportunities to ask questions about any detail of the study.

The protocol has been evaluated by the Ethical Committee in Clinical Investigation (CEIC) of the Research Institute of Primary Attention (IDAP) Jordi Gol and counts with its approval.

Confidentiality of the information: only the researchers will have access to the data of the subjects who accept. Once the study is finished, both the control group and the group intervened will be informed of the results; once the effectiveness of the Health Workshop is proven, they will be invited to participate.

## Discussion

The Health Workshop proposed in the present study is a different approach to Health Promotion, placing the emphasis on self responsibly of the persons being cared for.

The weekly cadence of a session during 8 weeks with recommendations of activities and exercises for the week as well as group support is an opportunity to incorporate healthy habits and make a commitment to self-care.

The hand outs as a reminder and reference provide a Health Manual to consult once the Workshop is over.

## Competing interests

The authors declare that they have no competing interests.

## Authors' contributions

Maria Antonia Campo, José Luis Del Val, Carolina Lapena, Francesc Orfila, participated in the revision of the bibliography and design of the study methodology. María Teresa Tierno and Enriqueta Borrás contributed in the writing of the present manuscript. Maria Antonia Campo, Carolina Lapena, Vicencia Laguna, Araceli García, Olga Lozano, Ziortza Martín and Rómulo are conducting the intervention groups. All of the authors have read and approved of the present manuscript.

## Pre-publication history

The pre-publication history for this paper can be accessed here:

http://www.biomedcentral.com/1471-2458/12/209/prepub
